# Relationship Between Sperm Parameters and Indices of Chromatin Condensation and DNA Fragmentation in Semen

**DOI:** 10.3390/biology14111550

**Published:** 2025-11-05

**Authors:** Othmane Adli, Noureddine Louanjli, Rachid Aboutaieb

**Affiliations:** 1 Laboratory of Sexual and Reproductive Health, Faculty of Medicine and Pharmacy, Hassan II University, Casablanca 20250, Morocco; raboutaieb@hotmail.fr; 2 Labomac IVF Centers and Clinical Laboratory Medicine, Casablanca 20100, Morocco; n.louanjli@gmail.com; 3 Department of Urology, Ibn Rochd University Hospital Center, Casablanca 20503, Morocco

**Keywords:** male infertility, sperm DNA fragmentation, sperm chromatin decondensation, teratozoospermia, sperm parameters

## Abstract

Infertility is a major health problem affecting about 15% of couples worldwide. Male etiology is found in almost one-third of cases. This study identified the nature of the relationship between sperm DNA fragmentation (SDF), sperm chromatin condensation (SCC) and sperm parameters. In this study, 80 samples were analyzed using two methods: the TUNEL technique to assess sperm DNA quality and aniline blue coloration to determine the level of chromatic condensation of spermatozoa. In addition, to specify the standard sperm parameters, the spermogram and the spermocytogram were analyzed. The main results revealed a significant difference between SDF and motility and, similarly, between SCC, motility, and teratozoospermia macrocephaly types (*p* < 0.0001, *p* < 0.0001, respectively), but no differences between SCC, SDF, and the other sperm parameters (*p* > 0.99).

## Simple Summary

Infertility is a major global health issue, affecting about 15% of couples, with male factors responsible for nearly one third of cases. Current evaluations usually rely on semen analysis, which measures sperm concentration, movement, and shape. While important, these tests often fail to explain why many men remain infertile. This limitation has led researchers to explore deeper aspects of sperm quality, particularly the condition of the genetic material it carries. In this study, we examined two hidden features: whether sperm DNA is damaged and whether it is properly condensed and packaged. We analyzed samples from 80 men and compared these factors with standard sperm characteristics. The results revealed that men with high DNA damage or poor chromatin packaging often had sperm with reduced motility and abnormal forms. This shows that conventional semen tests alone may not fully capture the causes of male infertility. Our findings emphasize the need to integrate DNA integrity and chromatin assessments into standard diagnostic practice. Such approaches would improve diagnostic precision, guide better treatment choices in assisted reproduction, and increase the chances of pregnancy for couples. In the long term, this knowledge will also support continued progress in reproductive medicine.

## 1. Introduction

Infertility is a disorder of the male or female reproductive system, characterized by the inability to achieve pregnancy after 12 months or more of regular, unprotected sexual intercourse, as defined by the World Health Organization (WHO). This condition may result from male, female, or unexplained factors, and some causes are preventable. Treatment often involves in vitro fertilization (IVF) and other assisted reproductive technologies (ART) [1].

In males, fertility primarily relies on spermatogenesis, a complex biological process in the testes that ensures the continuous and differentiated production of spermatozoa from immature germ cells. Spermatogenesis plays a crucial role in male reproductive capacity because it is regulated by hormonal factors and influenced by genetic and environmental conditions. Any disruption of this process can lead to quantitative or qualitative alterations in spermatozoa, resulting in infertility [2].

Male infertility can be categorized into nonidiopathic and idiopathic types. Nonidiopathic infertility includes identifiable causes and is divided into obstructive (e.g., blockages like congenital absence of the vas deferens due to CFTR mutations or acquired causes such as infections or vasectomy) and nonobstructive forms (e.g., impaired spermatogenesis caused by chromosomal abnormalities, hormonal imbalances, or environmental toxins) [3,4].

Idiopathic infertility refers to cases where no clear cause is found despite extensive evaluation. It is often associated with complex molecular mechanisms, unknown environmental factors, or oxidative stress affecting spermatogenesis.

One of the major factors contributing to both idiopathic and nonidiopathic male infertility is oxidative stress, which reflects an imbalance between reactive oxygen species (ROS) production and antioxidant defenses. While low levels of ROS are essential for normal sperm function, excessive ROS can impair sperm motility, damage DNA, and disrupt membrane integrity. These effects are particularly relevant in idiopathic infertility, where conventional analyses may not reveal underlying causes. As such, oxidative stress is considered a key mechanism contributing to sperm DNA fragmentation and chromatin defects, parameters that are assessed by SDF and SCC tests and may offer valuable diagnostic insight beyond standard semen analysis [5].

Advanced tests such as SDF and SCC analyses have become essential tools in evaluating the genetic integrity of sperm and understanding idiopathic cases of male infertility. The SDF test assesses the extent of DNA strand breaks in spermatozoa, with high fragmentation levels being associated with reduced fertilization capacity, impaired embryonic development, and increased miscarriage rates. In contrast, sperm chromatin decondensation assessment, particularly through the sperm chromatin condensation test, provides valuable insights into the integrity of the male genome, which is essential for successful fertilization and embryonic development [6]. During spermiogenesis, the process of chromatin remodeling occurs where the histone-based chromatin structure is replaced with a protamine-based structure (especially P2), leading to a highly condensed state within the sperm nucleus [7]. This condensation is crucial for protecting the DNA during its journey to the oocyte and ensuring proper packaging for fertilization. Methods such as terminal deoxynucleotidyl transferase dUTP nick end labeling (TUNEL) for SDF and aniline blue staining for SCC provide detailed insights into sperm chromatin status. These alterations are often linked to oxidative stress and cellular dysfunction. While first-line evaluations like spermograms and spermocytograms remain foundational, integrating these molecular-level analyses into routine diagnostic protocols could enhance the personalization of infertility management and improve the planning of assisted reproductive techniques [8].

Therefore, in this study, we selected the TUNEL and aniline blue assays to specifically evaluate DNA fragmentation and chromatin condensation defects, which are key indicators of sperm nuclear integrity and closely aligned with our study objectives.

We hypothesized that increased sperm DNA fragmentation and chromatin decondensation are significantly associated with altered conventional sperm parameters, reflecting a decline in sperm quality.

The objective of this study was to examine the relationships between sperm DNA fragmentation, sperm chromatin decondensation, and common sperm parameters.

## 2. Materials and Methods

### 2.1. Samples

This study was conducted on a population of 80 men of reproductive age who visited the reproductive biology laboratory (LABOMAC) between January 2024 and May 2024 to undergo sperm analysis tests for management as part of the exploration of couple infertility or an attempt at assisted reproductive technology through intrauterine insemination (IUI), in vitro fertilization (IVF), or intracytoplasmic sperm injection (ICSI).

The sperm samples were collected by masturbation after a period of sexual abstinence of 3 to 7 days into sterile containers, accompanied by a patient information sheet, in compliance with the necessary rules for aseptic sperm collection.

### 2.2. The Inclusion and Exclusion Criteria

In this study, eligible participants were men presented for an initial evaluation of primary infertility, defined by the absence of any prior conception, and capable of providing an ejaculated semen sample for analysis. To ensure cohort homogeneity and minimize potential confounding factors, the following exclusion criteria were applied: azoospermia (complete absence of sperm in the ejaculate); any form of obstructive infertility such as secondary infertility (defined by a history of prior conception); and clinically diagnosed varicocele, a vascular condition known to impair spermatogenesis. These criteria were established to specifically select cases of primary male infertility of non-obstructive and non-vascular origin.

### 2.3. Basic Sample Treatment

After sample collection, the samples were placed in an incubator at 35 °C for a liquefaction period of at least 30 min before any sperm processing. Then, conventional sperm parameters were measured via sperm analysis.

### 2.4. Sperm Parameter Analysis

#### 2.4.1. Sperm Analysis

The sperm analysis is used to quantify pH, volume (in mL), sperm concentration (in millions/mL), total sperm count (in millions/ejaculate), and sperm viability (in %), and to qualify motility (a; b; c). The analyzed sperm parameters include appearance, which is normally whitish or yellowish in the case of infection and/or suggestive of hematospermia in the case of the presence of red blood cells in the sperm [9].

The pH was measured after 30 min using a pH paper strip to avoid alkalinization, which increased over time. The volume was measured using a graduated tube. To assess motility, 10 μL of sperm were taken after homogenization and observed between a slide and coverslip under a microscope at 40× magnification, followed by a calculation of the percentage of each motility category (Table A1) [9].

Vitality is evaluated by mixing the sperm with eosin or nigrosin and observing them under a microscope at 40× magnification, where dead spermatozoa are stained red–pink, whereas live spermatozoa remain colorless. The concentration is determined by placing a drop of diluted sperm on a Malassez cell and counting it according to the formula N = (n) × the dilution factor × (y) × 1000, where N is the sperm concentration, n is the number of sperm counted, and y is the number of squares counted (total 100) [9]. The thresholds for the different sperm analysis parameters were established according to WHO standards (Table A2).

#### 2.4.2. Assessment of Sperm Morphology

The measurement of conventional sperm parameters via spermocytogram consists of the cytological examination of human spermatozoa, including the morphological analysis of abnormalities, the teratozoospermia index (TZI), and particularly the percentage of typical forms (FTs).

After homogenization of the sperm, a sperm smear is made and fixed with 1% ethanol. The slides were first immersed in hematoxylin for 1 to 3 min and then rinsed with water. The sample was then immersed in Shorr for 1–3 min, rinsed again with water, and finally dried on a heating plate to prepare for reading.

The smear is read under an optical microscope after immersion oil is added, with a magnification of 100×, or a computer-assisted sperm analysis system (CASA) is used to determine the various abnormalities of the spermatozoa.

For result analysis, the modified David classification was used to assess the presence or absence of abnormalities in different parts of the spermatozoa [10]. This assessment was conducted through a detailed spermocytogram, allowing classification of anomalies into specific groups based on the affected structure. Head defects included macrocephalic and microcephalic forms, as well as malformed acrosomes. Abnormalities of the flagellum and the midpiece, such as coiled, multiple, or irregular tails and midpiece deformities, were also recorded. This grouping enabled a comprehensive analysis of morphological integrity according to standardized criteria. The TZI percentage, which represents the average number of abnormalities per abnormal spermatozoon, is calculated on the basis of the values of the various spermocytogram parameters. Its theoretical maximum value is 4, and the typical forms correspond to Kruger’s criteria [11]. For the evaluation of sperm morphology, a normal threshold of FT > 4% was used [9,10,11].

### 2.5. Analysis of the Quality of the Genetic Material in Spermatozoa

The analysis of sperm quality alterations in this study focused on damage at the sperm nucleus level, which can be chromatin-related or DNA-related, in the context of male infertility.

#### 2.5.1. Index of SDF by the TUNEL Technique

The protocol for the TUNEL technique includes several sample preparation steps. First, fixation and permeabilization are performed by mixing the sperm with a modified PBS solution, followed by centrifugation and collection of the pellet. A drop of this pellet is used to prepare a smear, which is then dried on a heating plate at 36 °C. Next, a fixation solution (37 °C formaldehyde + PBS) was added, and the slides were left at room temperature for 30 min. After the samples were rinsed with PBS, drops of permeabilization solution (Triton X + citrate + distilled water) were added, and the samples were incubated at room temperature for 1 min. The slides were then rinsed again with a PBS solution and left to dry.

The labeling solution containing the TUNEL reaction mixture (In Situ Cell Death Detection Fluorescein, Roche^®^ Diagnostics GmbH, Mannheim, Germany) was then applied to the smears. The slides were covered and placed in a dark box, followed by incubation at 37 °C for 45 min. After rinsing with a modified PBS solution, drops of glycerol were added between the slides and coverslips (all steps were carried out in the dark).

#### 2.5.2. Index of SCC by Aniline Blue Staining

The protocol includes the following steps: fixation and permeabilization of the spermatozoa, using the same protocol as for sperm DNA fragmentation. Then, aniline blue staining was performed by applying 1 mL of stain to the smear for 15 min at room temperature, followed by rinsing the slides with tap water and drying.

### 2.6. Statistical Analysis

Statistical analyses were conducted using GraphPad Prism 10. The normality of data distribution was assessed using the Shapiro–Wilk test, and the homogeneity of variances was tested with Bartlett’s test. One-way ANOVA was applied to compare group means, followed by Bonferroni post hoc tests for multiple comparisons. No covariates were included in the model, as the study design did not involve confounding variables requiring adjustment. The results are expressed as the mean ± standard error of the mean (SEM), with a significance threshold set at *p* < 0.05.

## 3. Results

### 3.1. Sperm Parameter Analysis: Spermogram and Spermocytogram

**Figure 1:**
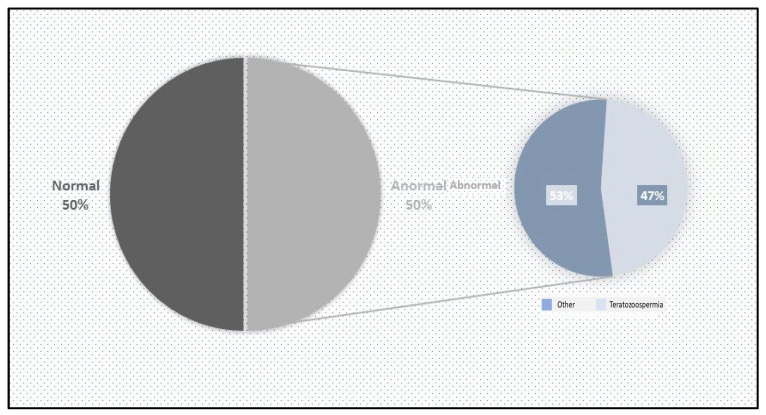
Graphical distribution of the samples.

A total of 80 patients of reproductive age were included in this study over a period of two months. Of these, 40 (50%) were included in the control group. This group had a percentage of typical forms (FTs) greater than 4%. The standard sperm parameters were satisfactory: viability above 50%, a sperm concentration over 16 million/mL, and motility above 40%. Additionally, the genetic material profile was normal, with fewer than 30% abnormalities based on sperm DNA fragmentation and condensation indices (Figure 1).

**Figure 2:**
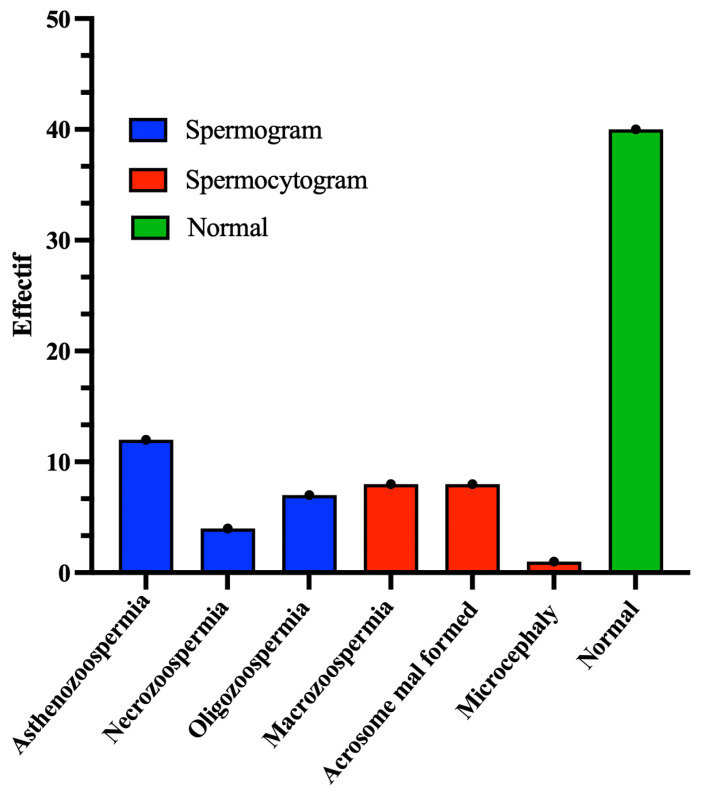
Illustration of the numbers of spermatic anomalies identified by tests of the spermatic parameters (spermograms and spermocytograms are represented in blue and red, respectively). Normal cases are shown in green.

The remaining 40 (50%) patients were classified as having abnormal sperm. Among them, 17 (42.5%) presented sperm abnormalities in morphology (monomorphe anomaly), characterized by sperm parameters with TZIs ranging between 1 and 4 and a percentage of typical forms (FTs) less than 4%. The other 23 (57.5%) exhibited abnormal sperm parameters, with vitality below 50% and/or a sperm count below 16 million/mL and/or sperm motility below 40% (Figure 2).

### 3.2. Relationship Between Genetic Material Quality and Patient Groups

#### 3.2.1. Relationship Between Sperm DNA Fragmentation and Patient Groups

**Figure 3:**
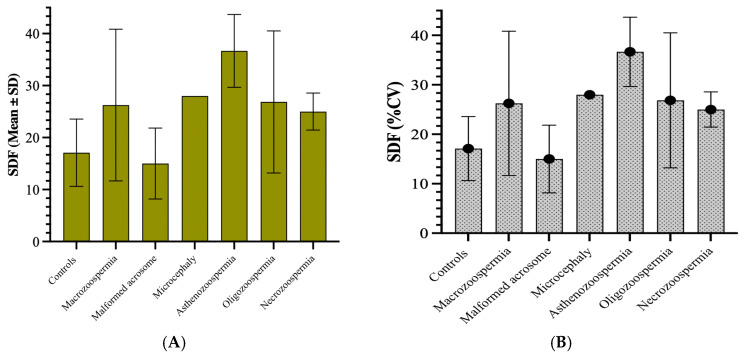
(**A**) Sperm DNA fragmentation levels presented as mean ± standard deviation across the different patient groups. (**B**) Coefficient of variation (%CV) of sperm DNA fragmentation among the same groups.

In the group of 40 patients whose sperm abnormalities were detected by spermocytogram and/or spermogram, 25 samples (62.5%) exhibited normal DNA fragmentation (SDF ≤ 30%), whereas the remaining 15 patients (37.5%) presented with elevated DNA fragmentation (SDF > 30%) (Figure 3).

The highest average values of sperm DNA fragmentation were observed in the asthenozoospermia group (36.67) and the necrozoospermia group (28.00). This indicates higher levels of fragmentation in these groups than in the other groups. The standard deviations varied significantly, with the asthenozoospermia group showing high variability (standard deviation of 6.998). In contrast, the microcephaly group showed no variability (standard deviation of 0), likely due to its small sample size (n = 1) (Figure 3A). The coefficient of variation (CV) revealed that the macrozoospermia group presented the highest relative variation (55.57%), followed by the oligozoospermia group (50.85%). This suggests a greater spread of values around the mean in these groups than in the other groups. In contrast, the microcephalic group showed no variation due to its single sample size (Figure 3B).

**Figure 4:**
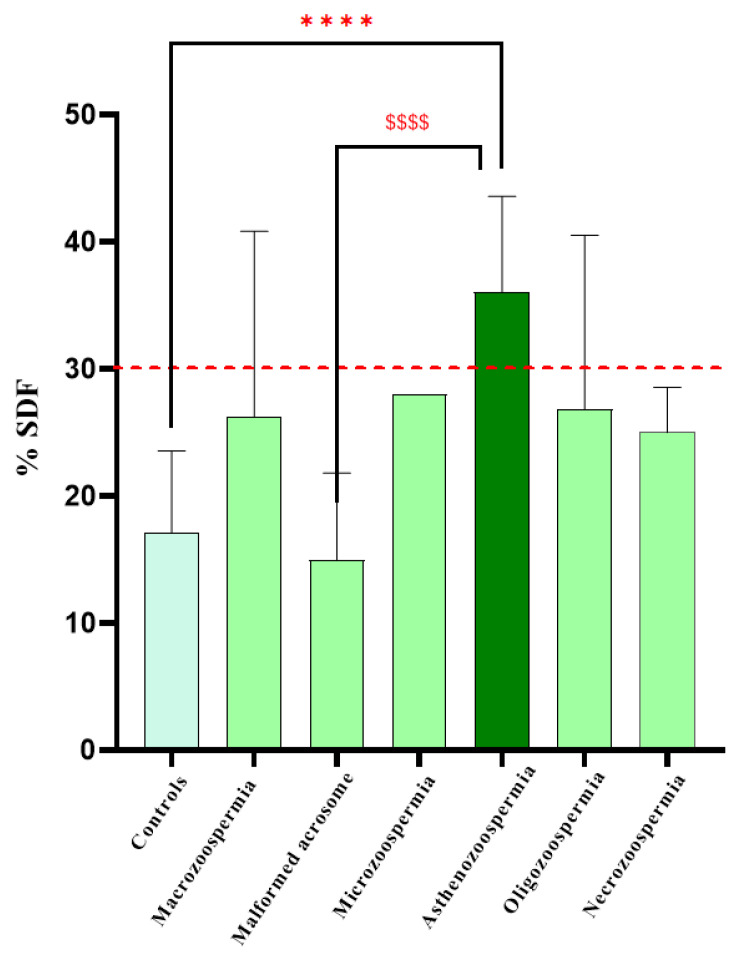
Graphical distribution of patient groups according to SDF percentage. Data analysis via one-way ANOVA revealed a significant difference between the groups. **** *p* < 0.0001 vs. Controls, $$$$ *p* < 0.0001 vs. Malformed acrosome. The red line represents the normal threshold, which is equal to 30%. One-way ANOVA revealed a significant effect of DNA fragmentation on spermatic anomalies (F6, 73 = 9.587, *p* < 0.0001). Bonferroni post hoc analysis revealed significant differences between the control and asthenozoospermia groups (*p* < 0.0001) and between the asthenozoospermia and malformed acrosome groups (*p* < 0.0001). However, there were no significant differences between the other groups.

One-way ANOVA revealed a significant effect of DNA fragmentation on spermatic anomalies (F6, 73 = 9.587, *p* < 0.0001). Bonferroni post hoc analysis revealed significant differences between the control and asthenozoospermia groups (*p* < 0.0001) and between the asthenozoospermia and malformed acrosome groups (*p* < 0.0001). However, there were no significant differences between the other groups (Figure 4).

#### 3.2.2. Relationship Between Sperm Chromatin Condensation and Patient Groups

**Figure 5:**
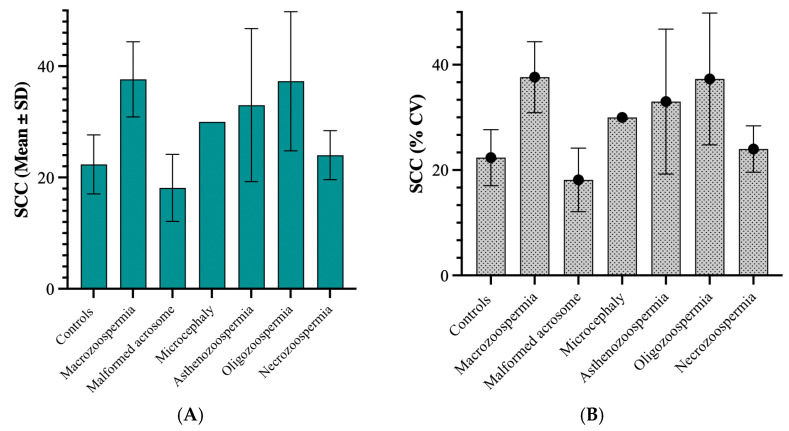
(**A**) Sperm chromatin condensation levels presented as mean ± standard deviation across the different patient groups. (**B**) Coefficient of variation (%CV) of SCC among the same groups.

Among the group of 40 patients with sperm abnormalities identified through spermocytogram and/or spermogram analysis, 22 samples (55%) exhibited condensed or normal chromatin (SCC ≤ 30%), whereas the remaining 18 patients (45%) presented decondensed chromatin (SCC > 30%) (Figure 5).

On average, the macrozoospermia and oligozoospermia groups presented the highest values, at 37.63 and 37.29, respectively, indicating elevated levels of sperm chromatin decondensation compared with the other groups. The standard deviations differed markedly across groups, with the asthenozoospermia group showing a standard deviation of 13.76, suggesting notable variability within this group. In contrast, the microcephaly group displayed no variation (standard deviation of 0), likely because its sample size was n = 1. The coefficient of variation (CV) test revealed that the asthenozoospermia group presented relative variation (41.69%), followed by the oligozoospermia group (33.56%). This suggests a wider spread of values around the mean in these groups than in the other groups. In contrast, the macrozoospermia group showed no variation due to its single sample size. The 75th percentile across all groups was well above the median, indicating a positive skew in the data distribution for these groups.

**Figure 6:**
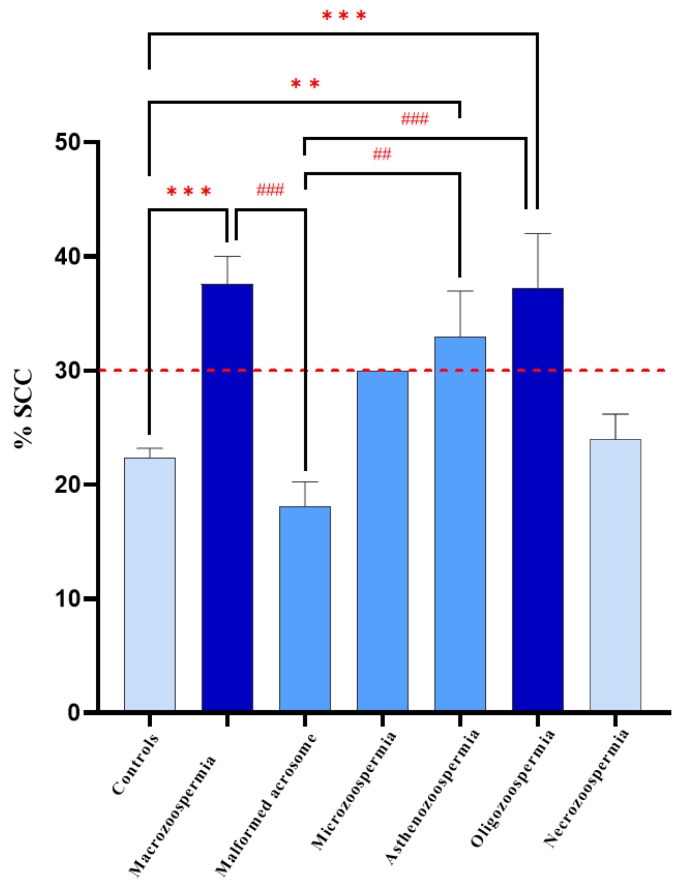
Graphical distribution of patient groups according to SCC percentage. Data analysis via one-way ANOVA revealed a significant difference between the groups. ** *p* < 0.01, *** *p* < 0.001, vs. Controls, and ## *p* < 0.01, ### *p* < 0.001 vs. Macrozoospermia. The red line represents the normal threshold, which is equal to 30%.

One-way ANOVA revealed a significant effect of DNA decondensation on spermatic anomalies (F6, 73 = 9.219, *p* < 0.0001). Bonferroni post hoc test revealed significant differences between the macrozoospermia (*p* = 0.0001), asthenozoospermia (*p* = 0.0030), and oligozoospermia (*p* = 0.0005) groups and the control group. Additionally, there was a significant difference between the macrozoospermia (*p* = 0.0002), asthenozoospermia (*p* = 0.0027), and oligozoospermia (*p* = 0.0004) groups and the malformed acrosome group. However, no significant differences were found between the other groups (*p* > 0.99) (Figure 6).

## 4. Discussion

The objective of this study was to investigate the interplay between sperm DNA fragmentation, sperm chromatin condensation, and conventional sperm parameters assessed through standard semen analysis. A total of 80 male patients undergoing infertility assessment were enrolled, aiming to explore the relationship between sperm nuclear quality and sperm parameters. SDF was measured using the TUNEL assay, while SCC was assessed using aniline blue staining.

Our results revealed statistically significant differences across several sperm parameters, underscoring the importance of integrating nuclear quality tests (SDF and SCC) into routine male fertility evaluations. Traditional semen analyses (spermogram, spermocytogram), although informative, do not reflect the full extent of sperm nuclear integrity, which plays a critical role in fertilization and embryonic development [12,13].

Interestingly, some abnormalities identified via basic tests such as asthenozoospermia and teratozoospermia showed strong associations with either fragmentation or decondensation indices. These associations appeared to be indirect and multifactorial, as supported in the literature [10]. In some patients, anomalies were reflected in both nuclear indices (SDF > 30% and SCC > 30%), suggesting a cumulative effect of multiple pathological processes on sperm quality. This cumulative effect has been explained in studies on the influence of patient age on DNA fragmentation [14], although age is not the only contributing factor. Furthermore, additional mechanisms, including apoptosis, damage during chromatin packing in spermiogenesis, and oxygen radical–induced DNA damage by reactive oxygen species (ROS), also contribute significantly to sperm nuclear impairment [15].

The control group exhibited relatively low levels of DNA fragmentation (17.1 ± 6.46) and chromatin decondensation (22.35 ± 5.30) compared to groups with abnormal semen parameters. This sharp contrast emphasizes the relation between sperm abnormalities and nuclear quality and supports previous findings highlighting the impact of oxidative stress and abnormal spermatogenesis on DNA integrity [14,16,17].

### 4.1. Sperm DNA Fragmentation

Sperm DNA carries paternal genetic information, and its structural integrity is essential for fertilization and embryo viability [18]. DNA damage can be induced by intrinsic factors such as defective chromatin packaging, and by extrinsic factors such as oxidative stress and environmental toxins. Under oxidative conditions, reactive oxygen species (ROS) can induce single- and double-strand breaks in DNA, thereby compromising sperm function [15].

In our study, a significant increase in SDF was found in patients with asthenozoospermia compared to the control group (*p* < 0.0001). The 12 patients with asthenozoospermia (30% of the cohort) exhibited sperm FT values > 4% and motility < 32%. This alteration in DNA can be explained by the spermatozoa’s microenvironment, which is rich in free radicals that induce oxidative stress and directly degrade the DNA. Furthermore, the presence of elevated SDF in sperm with asthenozoospermia suggests a dual impairment: reduced mitochondrial activity required for motility and increased oxidative damage affecting both the membrane and nuclear DNA [19]. As shown in the study by [20], reactive oxygen species, whether generated by leukocytes within the reproductive tract, mitochondrial dysfunction, or exposure to environmental toxins, produce oxidative lesions on nitrogenous bases and cleave the sugar–phosphate backbone, ultimately undermining chromatin compaction and compromising nuclear integrity. Moreover, mitochondrial ROS production itself has been identified as a key driver of both motility loss and DNA fragmentation, reinforcing the notion that oxidative stress sits at the crossroads of these two pathological processes [14]. Similar to our findings, Liu et al. conducted a correlation analysis between sperm DNA fragmentation and conventional semen parameters in a cohort of 101 infertile men. They demonstrated a significant correlation between SDF and three key functional metrics; sperm survival rate, sperm concentration, and the percentage of progressively motile sperm (*p* < 0.05 for all) [18].

### 4.2. Sperm Chromatin Condensation

The structural and functional organization of human spermatozoa is extremely complex and relies on a unique compaction system. The condensation of human sperm DNA is regulated by specific proteins that ensure the precise control of condensation and decondensation over time [16]. At certain stages of embryonic development, DNA must be decondensed to allow protein synthesis, while at other times, it must be condensed to protect it from degradation and damage [21]. Any disruption in this process can lead to decondensed chromatin, making DNA more vulnerable to damage. This delicate balance is maintained by the structural organization of DNA [17].

In this study, we observed a significant increase in chromatin decondensation in patients with macrozoospermia compared to controls (*p* < 0.00001). This subgroup (20% of altered patients) showed a high percentage of monomorphic anomalies with FT < 4%. Macrozoospermia often reflects defects in chromosomal segregation and is coupled with insufficient protamine incorporation. Throughout spermiogenesis, histone-bound chromatin is progressively exchanged for protamines, chiefly protamine 2, driving the extreme nuclear condensation characteristic of mature spermatozoa [22,23]. The intricate process of spermiogenesis, where haploid round spermatids metamorphose into spermatozoa, involves substantial cytoplasmic reduction and chromatin remodeling, resulting in a significant decrease in nuclear volume [24]. This decrease in nuclear volume, coupled with chromatin condensation, is essential for sperm motility, protection of the paternal genome, and successful fertilization [25]. Moreover, mutations in the AURKC gene disrupt sperm chromatin during spermatogenesis, as demonstrated by Ben Khelifa et al. (2011) [26] and Dieterich et al. (2007) [27], who discovered a novel homozygous AURKC mutation responsible for macrozoospermia and showed that loss of aurora kinase C activity impairs histone removal and protamine incorporation. Oxidative stress further compromises sperm nuclear integrity by preventing proper protamine cross-linking and inducing premature chromatin unpacking [8]. Consistent with our findings, Dadoune et al. (1988) showed that macrocephalic spermatozoa had a markedly low proportion of unstained heads (14%), reflecting pronounced DNA decondensation and thus corroborating the strong correlation we observed between head enlargement and chromatin decondensation [28].

### 4.3. Interplay Between SDF and SCC

Interestingly, while SDF and SCC represent distinct processes (strand breakage vs. compaction failure), they are often interrelated. Incomplete chromatin condensation has been proposed as a precursor to DNA fragmentation, as loosely packed DNA is more susceptible to ROS and endonuclease attack [14,29]. In our study, patients with elevated SCC often exhibited high SDF, suggesting that impaired chromatin packaging may predispose spermatozoa to fragmentation, a hypothesis supported by Larson-Cook et al. (2003) and Simon et al. (2017) [29,30]. This suggests that assessing both parameters provides a more comprehensive picture of nuclear sperm quality than either marker alone. However, the impact of DNA fragmentation and chromatin decondensation on ART outcomes remains controversial, with some studies finding no effect on fertilization or pregnancy rates [12,31], while others report improved ICSI success when selecting sperm with intact DNA and optimal chromatin compaction [32].

## 5. Limitations of the Study

This study presents several limitations that must be acknowledged. First, male infertility remains a sensitive and often taboo subject, especially in our country, Morocco, where cultural and societal norms can hinder open discussions and full participation. Moreover, the study was conducted in a private fertility center, which naturally limited the sample size and diversity, potentially affecting the generalizability of our findings. Due to this restricted setting and limited patient availability, long-term follow-up could not be achieved, which would have otherwise enriched the understanding of clinical outcomes.

Additionally, a subgroup of patients with rare conditions (microcephaly, n = 1) was excluded from statistical analyses due to insufficient sample size. While such cases may offer valuable descriptive insights, their exclusion was necessary to ensure the validity of our statistical models.

Furthermore, the relatively short data-collection period may limit the ability to account for potential temporal or seasonal variations in sperm parameters. Environmental and clinical confounding factors, such as exposure to toxins, infections, medication use, or oxidative stress, were not controlled in this study, which could influence sperm quality and represent a source of variability. Finally, although thresholds for parameters such as SDF and SCC were chosen based on the literature and clinical relevance, we acknowledge that some of these cut-off values may vary across studies and patient populations and thus could influence interpretation.

## 6. Conclusions

This study highlights the critical importance of evaluating sperm nuclear quality, specifically DNA fragmentation (SDF) and chromatin condensation (SCC), alongside conventional semen parameters in the assessment of male infertility. By examining a cohort of 40 patients with various sperm abnormalities versus 40 controls, we demonstrated significant differences between altered sperm parameters and elevated SDF and SCC values.

Notably, abnormalities such as asthenozoospermia and macrozoospermia were associated with distinct nuclear defects, emphasizing the diagnostic value of integrating SDF and SCC testing into routine fertility workups. Moreover, the observed interplay between chromatin decondensation and DNA fragmentation suggests that these two markers may reflect overlapping pathological mechanisms, potentially helping to orient assisted reproductive outcomes.

Ultimately, our results support the implementation of nuclear quality assays as main tools in clinical practice, with the potential to improve diagnostic precision, guide therapeutic decisions, and optimize patient management in male infertility.

## Data Availability

The datasets supporting the conclusions of this article are included within the article.

## Abbreviations

The following abbreviations are used in this manuscript:

ART	Assisted Reproductive Technology
AURKC	Aurora Kinase C
CASA	computer-assisted sperm analysis system
FTs	typical forms
ICSI	intracytoplasmic sperm injection
IUI	intrauterine insemination
IVF	in vitro fertilization
ROS	Reactive oxygen species
SCC	Sperm Chromatin Condensation
SDF	Sperm DNA Fragmentation
TZI	teratozoospermia index

## Appendix A

**Table A1:** Sperm motility parameters.

Type (a)	Rapid progressive
Type (b)	Nonprogressive
Type (c)	Immotile

**Table A2:** Standards for sperm.

Sperm Parameter	Threshold Values
Ejaculated volume	>1.4 mL
Sperm concentration (million/mL)	>16 million
Total sperm number (million/ejaculate)	>39 million
Total motility (progressive + nonprogressive)	>42%
Vitality	>58%
